# Rapid detection of rifampin resistance in *Mycobacterium tuberculosis* using nucleotide MALDI-TOF MS: a comparative study with phenotypic drug susceptibility testing and DNA sequencing

**DOI:** 10.1128/spectrum.00483-25

**Published:** 2025-05-30

**Authors:** Junxian Zhang, Haiyan Zhang, Jie Wang, Wenna Sun, Yan Liang, Yourong Yang, Qingwei Ma, Xueqiong Wu

**Affiliations:** 1 Beijing Key Laboratory of New Techniques of Tuberculosis Diagnosis and Treatment, Institute of Tuberculosis Research, Senior Department of Tuberculosis, The Eighth Medical Center of PLA General Hospital 12509, Beijing, China; 2 Bioyong Technologies Inc., Beijing, China; University of Hawaii at Manoa, Honolulu, Hawaii, USA

**Keywords:** rifampin resistance, *Mycobacterium tuberculosis*, nucleotide MALDI-TOF MS, drug susceptibility testing, *rpoB *mutations

## Abstract

Rifampin (RIF) resistance in *Mycobacterium tuberculosis* (*M.tb*) is primarily caused by mutations in the *rpoB* gene. Rapid and accurate detection of RIF resistance is critical for effective tuberculosis (TB) control. Nucleotide matrix-assisted laser desorption/ionization time-of-flight mass spectrometry (MALDI-TOF MS) is an emerging technology used to detect RIF resistance-associated *rpoB* mutations in 210 *M*.*tb* clinical isolates, including 107 RIF-sensitive and 103 RIF-resistant strains, as determined by phenotypic drug susceptibility testing (DST). DNA sequencing was used as the reference method to validate nucleotide MALDI-TOF MS results. Nucleotide MALDI-TOF MS demonstrated a sensitivity of 93.2%, specificity of 98.1%, and an overall accuracy of 95.7% compared to phenotypic DST. The Kappa value between nucleotide MALDI-TOF MS and phenotypic DST was 0.91, indicating excellent agreement. DNA sequencing confirmed that nucleotide MALDI-TOF MS successfully identified RIF resistance-associated mutations, particularly in codons 450, 445, and 435 of the *rpoB* gene. Among the 61 isolates analyzed by DNA sequencing, nucleotide MALDI-TOF MS and sequencing results were consistent for 52 of 56 RIF-resistant strains and all five RIF-sensitive strains, with an overall concordance of 93.4%. Importantly, nucleotide MALDI-TOF MS accurately detected heteroresistance in eight isolates (14.3%), confirmed by sequencing. These results support that nucleotide MALDI-TOF MS is a rapid, accurate, and reliable method for detecting *rpoB* mutations associated with RIF resistance in *M.tb*. Its high concordance with DNA sequencing, excellent diagnostic performance, and ability to identify heteroresistance highlight its potential as a valuable tool for early TB diagnostics and improve the precision of chemotherapy regimen development.

**IMPORTANCE:**

The emergence of multidrug-resistant tuberculosis (MDR-TB) and rifampin-resistant tuberculosis (RR-TB) poses a significant challenge to global tuberculosis (TB) control efforts. Rifampin (RIF) resistance is a critical marker for MDR-TB, which requires more complex, prolonged, and costly treatment regimens. Early and accurate detection of RIF resistance is crucial for effective TB control. This study evaluates the performance of nucleotide MALDI-TOF MS, an innovative technology, for detecting RIF resistance-associated mutations in the rpoB gene. The method demonstrates high sensitivity (93.2%) and specificity (98.1%), with the added advantage of identifying heteroresistance, capabilities that are lacking in conventional methods. These capabilities are crucial for early diagnosis, guiding personalized treatment regimens, and curbing the transmission of drug-resistant TB. The findings demonstrate that nucleotide MALDI-TOF MS provides a rapid, high-throughput, and cost-effective alternative for detecting rpoB gene mutations associated with RIF resistance.

## INTRODUCTION

Tuberculosis (TB) remains a major global health challenge, with an estimated 10.8 million new cases and 1.25 million deaths reported in 2023 alone (1). A particularly concerning aspect of TB is the rise of drug-resistant strains. In 2022, approximately 410,000 people worldwide suffered from multidrug-resistant/rifampicin-resistant TB (MDR/RR-TB), but only about two in five of these patients were diagnosed and included in treatment (2). Rifampin (RIF), a first-line anti-tuberculosis drug, is a cornerstone of TB treatment regimens. In China, 8.2% of clinical isolates of *Mycobacterium tuberculosis* (*M.tb*) are resistant to RIF (3). However, resistance to RIF often signals broader drug resistance as it is strongly associated with resistance to other first-line drugs, such as isoniazid (3, 4). In fact, 83.1%, 26.6%, and 3.2% of RR-TB cases are MDR-TB, pre-extensively drug-resistant tuberculosis (pre-XDR-TB), and extensively drug-resistant tuberculosis (XDR-TB), respectively. Additionally, 30.6% of RR-TB strains have been identified as resistant to pyrazinamide (5). RR-TB patients are usually treated with the MDR-TB regimen. When RIF resistance is detected, second-line anti-TB drugs need to be used, significantly complicating treatment. The short regimen for RR-TB needs to last 6 or 9 months, and the longer regimen needs to take 18–20 months (6). This not only increases the duration and cost of treatment but also reduces its overall efficacy. Therefore, the rapid and accurate detection of drug resistance (especially RIF resistance) in *M.tb* isolates is crucial for timely initiation of effective treatment, improvement of patient outcomes, and prevention of further transmission.

Traditional phenotypic drug susceptibility testing (DST) and rapid genotypic DST (also referred to as molecular DST) methods are the primary approaches for clinical laboratory detection of drug-resistant TB (7). Phenotypic DST is considered the gold standard for determining *M.tb* drug susceptibility as it can detect the sensitivity and resistance level of multiple first- and second-line anti-TB drugs (7, 8). However, phenotypic DST has several limitations, including long turnaround times (ranging from weeks to months), technical complexity, and biosafety risks (9). In addition, the presence of heteroresistance, where both wild-type and mutant alleles coexist, further complicates resistance detection using conventional methods (10). These limitations hinder its ability to meet the demands of rapid clinical diagnosis and may delay the initiation of effective treatment.

With a deeper understanding of the molecular mechanism of drug resistance in *M.tb*, gene mutation detection technologies and molecular biology-based diagnostic products have emerged and been increasingly used in clinical practice (7). These advancements have significantly reduced detection time and improved diagnostic efficiency. To improve the accessibility and diagnostic efficiency of drug-resistant TB, the World Health Organization (WHO) recommends the use of various rapid molecular DST methods to detect specific mutations known to be associated with phenotypic drug resistance. It has also affirmed the feasibility of rapid molecular testing for resistance to rifampicin (RIF), isoniazid (INH), and fluoroquinolones (FQ) (7). Despite these advancements, current molecular DST methods have both advantages and limitations. Linear probes and gene chip methods provide insights into common mutation sites and characteristics of drug-resistance genes, but their hybridization and color development processes are cumbersome and time-consuming (11, 12). Moreover, the open detection format increases the risk of amplified product contamination, potentially leading to false-positive reports of drug resistance. Real-time fluorescence quantitative polymerase chain reaction (qPCR) and PCR-probe melting curve methods employ closed-tube systems that eliminate cross-contamination and simplify the detection process, achieving results in just 2–3 hours (13, 14). However, these methods can only identify the presence of drug-resistant gene mutations without specific mutation sites and types. Since some gene mutations may not be directly related to drug resistance, these methods can yield false-positive results. In addition, they are limited in detecting heteroresistance (the coexistence of drug-susceptible and drug-resistant subpopulations). DNA sequencing remains the gold standard for molecular DST, offering high accuracy in identifying mutations associated with drug resistance (7). However, its application is limited by technical complexity and the need for specialized infrastructure. Currently, DNA sequencing is primarily conducted by sequencing companies and has yet to be widely implemented in clinical laboratories.

Matrix-assisted laser desorption/ionization time-of-flight mass spectrometry (MALDI-TOF MS) has been used as a microbial diagnostic technology (15). Recent advancements in mass spectrometry have introduced innovative methods for genotypic analysis, such as nucleotide MALDI-TOF MS, which provides a rapid and cost-effective alternative for detecting resistance mutations (16, 17). The basic principle involves charge transfer between the matrix and the sample, leading to sample ionization. The ionized sample travels through a vacuum tube under an electric field, and its detection is based on the time it takes to reach the detector. The mass-to-charge ratio (M/Z) of the ion is directly proportional to its flight time, enabling analysis of the ion, measurement of the molecular weight of the sample molecule, and subsequent determination of the analyte’s genotype (18). This technology offers several advantages, including high accuracy, strong flexibility, high throughput, short detection times, and high-cost performance (16, 17). In China, several biotechnology companies provide clinical testing services using nucleotide MALDI-TOF MS for *M.tb* and its resistance-related genotypic mutations (16, 17, 19). Chinese experts have also reached a consensus on the application of nucleotide MALDI-TOF MS in diagnosing TB and non-tuberculosis mycobacterium (NTM) diseases (20). However, the technical indicators of these companies’ products have not been publicly disclosed. Additionally, their products have not yet been approved by China’s National Medical Products Administration, and the standardization of their commercial services remains incomplete. More research is needed to validate its performance across diverse clinical and geographic settings.

Current research has demonstrated that mutations in the *rpoB* gene, particularly within the rifampin resistance-determining region (RRDR), are responsible for most of RIF resistance in *M.tb* (3, 21). Hotspot mutations in codons 450, 445, and 435 are most frequently associated with resistance, making them key targets for molecular diagnostics (3, 21). Previous studies have demonstrated the utility of nucleotide MALDI-TOF MS in detecting *rpoB* mutations (16–19, 22). However, most research in China has primarily utilized commercial nucleotide MALDI-TOF MS to analyze strains from eastern and western regions. There is a need for larger-scale and more extensive studies to evaluate the accuracy, sensitivity, and specificity of nucleotide MALDI-TOF MS in real-world clinical settings. Additionally, integrating nucleotide MALDI-TOF MS with other molecular and phenotypic methods can provide a more comprehensive understanding of TB drug resistance patterns and improve the diagnostic accuracy.

In this study, we aimed to develop a method that integrates PCR amplification, single-base extension reactions, and mass spectrometry detection to identify characteristic fragments of the *rpoB* gene associated with RIF resistance. Our objective is to determine the *rpoB* mutation genotypes of *M.tb* isolates and evaluate the diagnostic performance of nucleotide MALDI-TOF MS in detecting RIF resistance among a large number of *M.tb* clinical isolates from the north of China. We compared the results obtained using nucleotide MALDI-TOF MS with those from phenotypic DST and DNA sequencing to assess its sensitivity, specificity, and ability to detect heteroresistance. Our findings provide valuable insights into the utility of nucleotide MALDI-TOF MS as a rapid and accurate diagnostic tool for RIF resistance and highlight its potential to improve TB control efforts in high-burden settings.

## MATERIALS AND METHODS

### *M.tb* reference strains and clinical isolates

The *M.tb* H37Rv reference strain was obtained from the National Institutes for Food and Drug Control, Beijing, China. A total of 210 *M*.*tb* clinical isolates were collected from the PLA TB Specimen Resource Bank, the Eighth Medical Center of PLA General Hospital, Beijing, China. Among these, 107 strains were identified as rifampin (RIF)-sensitive and 103 as RIF-resistant based on phenotypic drug susceptibility testing (DST).

### Phenotypic drug susceptibility testing

Mycobacterial culture and phenotypic DST were performed according to the “Laboratory Science Procedure of Diagnostic Bacteriology in Tuberculosis” (1995 edition, Chinese Anti-Tuberculosis Association). The absolute concentration method on the Löwenstein-Jensen (L-J) medium was used to determine the drug susceptibility. RIF resistance was defined as bacterial growth on the medium containing RIF at a concentration of 50 µg/mL (23).

### DNA extraction

DNA extraction was performed as follows: a single colony of mycobacteria, cultured for 3 weeks on L-J medium, was transferred into a centrifuge tube containing 500 µL of TE buffer (10 mM Tris, 1 mM EDTA, pH 8.0). The sample was then inactivated at 80°C for 30 minutes. Lysozyme was added to a final concentration of 2 mg/mL, and the mixture was incubated at 37°C for 2 hours. Sodium dodecyl sulfate (SDS, final concentration 1%) and proteinase K (final concentration 50 µg/mL) were added, followed by incubation at 55°C for 2 hours. The lysates were extracted with phenol and DNA extraction solution (Solarbio Science & Technology Co., Ltd., Beijing, China). DNA was precipitated with 2 volumes of absolute ethanol and 1/10 vol of 3 M sodium acetate (pH 5.2), dissolved in TE buffer, and stored at −20°C. The concentration of DNA samples and the A260/A280 ratio were measured using a NanoPhotometer N60 UV spectrophotometer (IMPLEN GmbH, Germany). An A260/A280 ratio ≥1.8 indicates a low risk of protein contamination and confirms that the sample purity meets the requirements for downstream experiments. DNA samples were then diluted to a working concentration of 100 ng/µL with TE buffer.

### Nucleotide MALDI-TOF MS detection

Nucleotide MALDI-TOF MS was used to detect *M.tb* RIF-resistant genotypes. The experimental steps were as follows: (1) PCR amplification: The *rpoB* gene was amplified using the forward primer 5′-ACGTTGGATGGCACGCTCACGTGACAGAC-3′ and the reverse primer 5′-ACGTTGGATGCCGCGATCAAGGAGTTCTTC-3′ (synthesized by Generay Biotech Co., Ltd., Shanghai, China). Two reaction systems (W1 and W2) were prepared, each containing 2.8 µL reaction solution I (dNTPs, Tris-HCl, MgCl2), 0.2 µL enzyme I (amplification enzyme and UNG enzyme, Bioyong Technologies Inc., Beijing, China), 1 µL 0.5 µM primer mixture, and 1 µL DNA template. The PCR conditions were as follows: 50°C for 2 min; 95°C for 2 min; 45 cycles of (95°C for 30 s, 56°C for 30 s, and 72°C for 30 s); and 72°C for 5 min (2). Shrimp alkaline phosphatase (SAP) digestion: to remove residual dNTPs, 1.5 µL reaction solution II (Tris-HCl, MgCl2) and 0.5 µL enzyme II (SAP, Bioyong Technologies Inc., Beijing, China) were added to the PCR products, followed by incubation at 37°C for 30 min and 65°C for 5 min (3). Single-base extension (SBE): the SAP-digested products were subjected to SBE using 0.83 µL reaction solution III (ddNTPs, Tris-HCl, MgCl2), 0.23 µL enzyme III (extensase, Bioyong Technologies Inc., Beijing, China), and 0.94 µL 5 µM extension primer mixture (Table 1, synthesized by Generay Biotech Co., Ltd., Shanghai, China). The reaction conditions were as follows: 94°C for 30 s; 40–50 cycles of [94°C for 5 s, 5 cycles of (56°C for 5 s and 80°C for 5 s)]; and 72°C for 3 min (4). Purification and sampling: The SBE products were purified using 41 µL ultra-pure water and 15 mg resin (Bioyong Technologies Inc., Beijing, China). After centrifugation, 0.5 µL of the purified product was spotted onto a microarray chip (Bioyong Technologies Inc., Beijing, China) (5). Mass spectrometry detection and analysis: the SBE products were detected using the MassARRAY Analyzer 4 system. The extension products were analyzed based on their specific molecular weights to identify the *rpoB* mutation sites (shown in Table 2). Interpretation criteria were as follows: (i) If only one mass peak corresponding to the wild-type or mutant type was observed, the genotype was determined as the corresponding type. (ii) If peaks corresponding to both wild-type and mutant types were observed, it was identified as heteroresistance. (iii) If no peaks were observed for either the wild-type or mutant type, the experiment was deemed a failure.

**TABLE 1:** Single-base extension primer sequences and related information in nucleotide MALDI-TOF MS assay

Reaction system, tube	Name of primers	Single-base extension primers	Loci	Nucleotide change^*a*^
W2	TB-W2-1-UEP	TCTTCGGCACCAGCCAGC	430	C**T**G→C**C**G
W1	TB-W1-1-UEP	TTCGGCACCAGCCAGCTGAGC	432	**C**AA→**A**AA/**G**AA
W2	TB-W2-2-UEP	CCAGCCAGCTGAGCC	432	C**A**A→C**C**A
W1	TB-W1-2-UEP	CGGGTTGTTCTGGTCCATGAA	432	CA**A**→CA**T**
W1	TB-W1-3-UEP	CAGCTGAGCCAATTCATG	435	**G**AC→**T**AC
W2	TB-W2-3-UEP	ACAGCGGGTTGTTCTGG	435	G**A**C→G**T**C/G**G**C
W1	TB-W1-4-UEP	CGACAGCGGGTTGTTCTG	435	GA**C**→GA**G**
W1	TB-W1-5-UEP	CTGTCGGGGTTGACC	445	**C**AC→**G**AC/**T**AC/**A**AC
W1	TB-W1-6-UEP	CAGTCGGCGCTTG	445	C**A**C→C**T**C/C**G**C
W2	TB-W2-4-UEP	GTCGGGGTTGACCCA	445	CA**C**→CA**A**
W2	TB-W2-5-UEP	TGACCCACAAGCGCCGACTGT	450	T**C**G→T**T**G/T**G**G
W1	TB-W1-14-UEP1	ATCGCCGGGCCCCAGCGC	450	TC**G**→TC**C**
W2	TB-W2-6-UEP	CAGACCGCCGGGCCCC	452	C**T**G→C**C**G
				

^*a*^ Bold represents the specific nucleotide detected.

**TABLE 2:** Molecular weights of extension primers and extension products in nucleotide MALDI-TOF MS assay

Loci	Extension primer	MW of the extended primer	Genotype 1^*a*^ (wild-type)	MW^*b*^ of genotype 1 extension products	Genotype 2^*a*^ (mutant)	MW of genotype 2 extension products
430	TB-W2-1-UEP	5420.50	C**T**G	5747.60	C**C**G	5667.70
432	TB-W1-1-UEP	6392.20	**C**AA	6639.30	**A**AA	6663.40
432	TB-W1-1-UEP	6392.20	**C**AA	6639.30	**G**AA	6679.40
432	TB-W2-2-UEP1	4522.90	C**A**A	4794.20	C**C**A	4770.10
432	TB-W1-2-UEP	6468.20	CA**A**	6795.30	CA**T**	6739.40
435	TB-W1-3-UEP	5483.60	**G**AC	5770.80	**T**AC	5810.70
435	TB-W2-3-UEP	5257.40	G**A**C	5584.50	G**T**C	5528.60
435	TB-W2-3-UEP	5257.40	G**A**C	5584.50	G**G**C	5504.60
435	TB-W1-4-UEP	5546.60	GA**C**	5833.80	GA**G**	5793.80
445	TB-W1-5-UEP	4600.00	**C**AC	4847.20	**G**AC	4887.20
445	TB-W1-5-UEP	4600.00	**C**AC	4847.20	**T**AC	4927.10
445	TB-W1-5-UEP	4600.00	**C**AC	4847.20	**A**AC	4871.20
445	TB-W1-6-UEP	3966.60	C**A**C	4293.70	C**T**C	4237.80
445	TB-W1-6-UEP	3966.60	C**A**C	4293.70	C**G**C	4213.80
445	TB-W2-4-UEP	4609.00	CA**C**	4856.20	CA**A**	4880.20
450	TB-W2-5-UEP	6376.20	T**C**G	6623.30	T**T**G	6703.20
450	TB-W2-5-UEP	6376.20	T**C**G	6623.30	T**G**G	6663.40
450	TB-W1-14-UEP1	5446.67	TC**G**	5693.87	TC**C**	5733.87
452	TB-W2-6-UEP	4813.10	C**T**G	5084.30	C**C**G	5100.30

^*a*^ Bold represents the specific nucleotide detected.

^*b*^ MW represents the molecular weight.

### DNA sequencing

Sanger DNA sequencing was performed to validate the nucleotide MALDI-TOF MS results. The *rpoB* gene was amplified using forward primer 5′-GGTGGTCGCCGCGATCAAG-3′ and reverse primer 5′-CGAGCCGATCAGACCGATGT-3′ (synthesized by Sangon Biotech Co., Ltd., Shanghai, China). In a 50 uL reaction system, 25 uL 2 × Master Mix, 2 uL 10 uM forward primer, 2 uL 10 uM reverse primer, ＜1 µg DNA template, and ddH2O were added to a total of 50 uL. PCR conditions were as follows: 94°C for 3 min; 30 cycles of (94°C for 30 s, 55°C for 30 s, 72°C for 60 s); and 72°C for 5 min.

### Statistical analysis

Statistical analysis was performed using MedCalc v22.021 and SPSS 22.0 software. Sensitivity, specificity, positive predictive value (PPV), negative predictive value (NPV), accuracy, and AUC were calculated with MedCalc v22.021. SPSS 22.0 software was used to calculate kappa statistics. The Kappa test was used to evaluate the consistency between phenotypic DST and nucleotide MALDI-TOF MS results. Kappa values were interpreted as follows: <0.2 (poor agreement), 0.2–0.4 (fair agreement), 0.4–0.6 (moderate agreement), 0.6–0.8 (strong agreement), and 0.8–1.0 (excellent agreement).

## RESULTS

### Phenotypic DST and genotypic analysis of the *rpoB* gene

Phenotypic DST and nucleotide MALDI-TOF MS were used to analyze the drug susceptibility of 210 *M*.*tb* clinical isolates (shown in Tables 3 and 4). Of the 103 RIF-resistant strains, nucleotide MALDI-TOF MS identified 96 as resistant and seven as sensitive. Of the 107 RIF-sensitive strains, it identified 105 as sensitive and two as resistant. The sensitivity and specificity of nucleotide MALDI-TOF MS were 93.2% (95% CI: 86.5%–97.2%) and 98.1% (95% CI: 93.4%–99.8%), respectively. The positive predictive value, negative predictive value, and overall accuracy were 98.0%, 93.8%, and 95.7%, respectively (Table 3).

**TABLE 3:** Comparison of nucleotide MALDI-TOF MS and phenotypic DST for RIF resistance detection^*a*^

Method	DST		
	RIF-resistant	RIF-sensitive	Total
MS			
RIF-resistant	96	2	98
RIF-sensitive	7	105	112
Total	103	107	210

^*a*^ Performance metrics: Sensitivity: 93.2% (95% CI: 86.5%–97.2%) Specificity: 98.1% (95% CI: 93.4%–99.8%) accuracy: 95.7% (95% CI: 92.0%–98.0%) kappa Value: 0.91 AUC: 0.96.

**TABLE 4:** Identification of 210 *M*.*tb* clinical isolates by conventional DST and nucleotide MALDI-TOF MS

DST	Nucleotide MALDI-TOF MS detection^*a*^	No. of strains
RIF-resistant strains	T**C**G(Ser)450T**T**G(Leu)	54
(*n* = 103)	T**C**G(Ser)450T**G**G(Trp)	1
	TC**G**(Ser)450TC**C**(Ser)	1
	**C**AC(His) 445**T**AC(Tyr)	7
	**C**AC(His) 445**T**AC(Tyr),445WT	2
	**C**AC(His)445**G**AC(Asp)	2
	C**A**C(His)445C**T**C(Leu)	3
	C**A**C(His)445C**G**C(Arg)	4
	**C**AC(His)445**A**AC(Asn), C**A**C(His)445C**G**C(Arg)	2
	G**A**C(Asp)435G**T**C (Val)	5
	**G**AC(Asp)435**T**AC(Tyr)	5
	G**A**C (Asp)435G**G**C(Gly)	1
	C**T**G(Leu)452C**C**G(Pro)	4
	**C**AA(Gln)432**A**AA(Lys)	1
	C**T**G(Leu)430C**C**G(Pro), **G**AC(Asp)435**T**AC(Tyr)	1
	C**T**G(Leu)430C**C**G(Pro), G**A**C(Asp)435G**G**C(Gly)	2
	C**T**G(Leu)430C**C**G(Pro), **C**AC(His)445**A**AC(Asn)	1
	Wild type	7
RIF-sensitive strains	Wild type	105
(*n* = 107)	C**T**G(Leu)452C**C**G(Pro)	2
Total		210

^*a*^ Bold represents the specific nucleotide detected.

### Consistency between nucleotide MALDI-TOF MS and DNA sequencing

To validate the detection results of nucleotide MALDI-TOF MS, 61 clinical isolates were selected and further analyzed using nucleotide MALDI-TOF MS and DNA sequencing (Table 5). The results showed that nucleotide MALDI-TOF MS and DNA sequencing were consistent for 52 out of 56 RIF-resistant strains and for all five RIF-sensitive strains, with an overall concordance rate of 93.4%. Among nine isolates with discordant results between phenotypic DST and nucleotide MALDI-TOF MS (Table 4), the sequencing results were completely consistent with those of nucleotide MALDI-TOF MS.

**TABLE 5:** Identification of 61 *M*.*tb* clinical isolates by DST, nucleotide MALDI-TOF MS, and DNA sequencing

DST	Nucleotide MALDI-TOF MS^*a*^	DNA sequencing^*a*^	No. of strains
RIF-resistant strains	T**C**G(Ser)450T**T**G(Leu)	T**C**G(Ser)450T**T**G(Leu)	7
(*n* = 56)	T**C**G(Ser)450T**G**G(Trp)	T**C**G(Ser)450T**G**G(Trp)	1
	TC**G**(Ser)450TC**C**(Ser)	T**CG**(Ser)450T**AC**(Tyr)	1
	**C**AC(His)445**T**AC(Tyr)	**C**AC(His)445**T**AC(Tyr)	7
	**C**AC(His)445**T**AC(Tyr), 445 WT	**C**AC(His)445**T**AC(Tyr), 445 WT	2
	**C**AC(His)445**G**AC(Asp)	**C**AC(His)445**G**AC(Asp)	2
	C**A**C(His)445C**T**C(Leu)	C**A**C(His)445C**T**C(Leu)	3
	C**A**C(His)445C**G**C(Arg)	C**A**C(His)445C**G**C(Arg)	3
	C**A**C(His)445C**G**C(Arg)	C**A**C(His)445C**G**C(Arg), CA**G**(Gln)429CA**C**(His)	1
	**C**AC(His)445**A**AC(Asn), C**A**C(His)445C**G**C(Arg)	**CA**C(His) 445**AG**C(Ile)	2
	G**A**C(Asp)435G**T**C(Val)	G**A**C(Asp)435G**T**C(Val)	5
	**G**AC(Asp)435**T**AC(Tyr)	**G**AC(Asp)435**T**AC(Tyr)	4
	**G**AC(Asp)435**T**AC(Tyr)	GAC(Asp)435TAC(Tyr), AAC(Asn)437CAC(His)	1
	G**A**C(Asp)435G**G**C(Gly)	GAC(Asp)435GGC(Gly), C**T**G(Leu)430C**G**G(Arg)	1
	C**T**G(Leu)452C**C**G(Pro)	C**T**G(Leu)452C**C**G(Pro)	4
	**C**AA(Gln)432**A**AA(Lys)	**C**AA(Gln)432**A**AA(Lys)	1
	C**T**G(Leu)430C**C**G(Pro), **G**AC(Asp)435**T**AC(Tyr)	C**T**G(Leu)430C**C**G(Pro), **G**AC(Asp)435**T**AC(Tyr)	1
	C**T**G(Leu)430C**C**G(Pro), G**A**C(Asp)435G**G**C(Gly)	C**T**G(Leu)430C**C**G(Pro), G**A**C(Asp)435G**G**C(Gly)	2
	C**T**G(Leu)430C**C**G(Pro), **C**AC(His)445**A**AC(Asn)	C**T**G(Leu)430C**C**G(Pro), **C**AC(His)445**A**AC(Asn)	1
	Wild-type	Wild-type	7
RIF-sensitive strains	Wild-type	Wild-type	3
(*n* = 5)	C**T**G(Leu)452C**C**G(Pro)	C**T**G(Leu)452C**C**G(Pro)	2
Total			61

^*a*^ Bold represents the specific nucleotide detected.

### Detection of *rpoB* gene mutations

**Fig 1:**
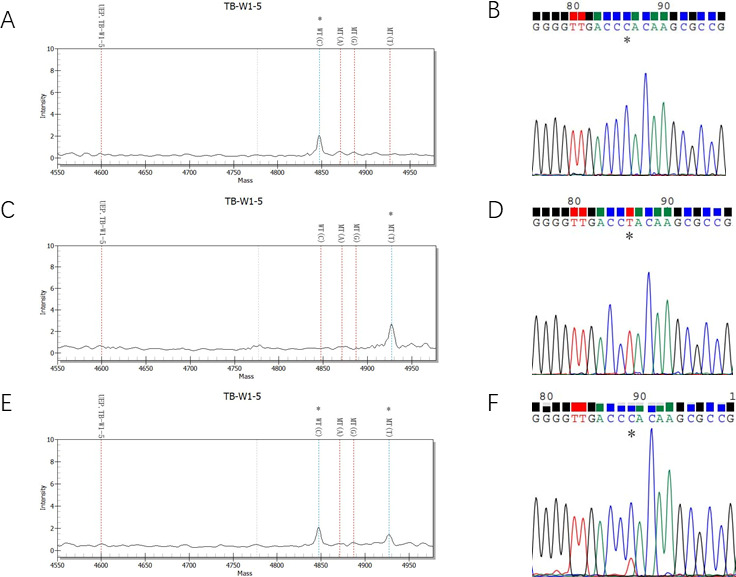
Comparison of nucleotide MALDI-TOF MS and Sanger sequencing results for *rpoB* mutations. A, B: MS and sequencing peaks for a RIF-sensitive isolate (codon 445-CAC). C, D: MS and sequencing peaks for a RIF-resistant isolate (codon 445-TAC). E, F: MS and sequencing peaks for a heteroresistant isolate (codon 445-TAC and codon 445-CAC ).

Nucleotide MALDI-TOF MS successfully identified multiple *rpoB* gene mutations associated with RIF resistance (Tables 4 and 5 and Fig. 1). Among the 103 RIF-resistant *rpoB* strains, 87 strains (84.5%) carried mutations in codons 450, 445, and 435. Codon 450 was the most common mutation site (56/103, 54.4%), with three mutation types, of which TCG (Ser) → TTG (Leu) was the most frequent (54/103, 52.4%). Codon 445 was the second-most common site (20/103, 19.4%) with five mutation types, and CAC (His) → TAC (Tyr) was the most frequent mutation type (9/103, 8.7%). Codon 435 ranked third (11/103, 10.7%) with three mutation types, with GAC (Asp) → TAC (Tyr) and GAC (Asp) → GTC (Val) being the most frequent (5/103, 4.9%). In codons 452, 450, 445, 435, 432, and 430, 14 mutation genotypes were identified, including 88 isolates with single-point mutations, two isolates with a single-point mutation and wild type (WT) at the same codon, four isolates with double-point mutations, and two isolates with two base mutations at the same codon.

### Detection of heteroresistance

Among the 56 phenotypically RIF-resistant isolates, heterogeneous resistance was identified in eight isolates (14.3%) through nucleotide MALDI-TOF MS and gene sequencing (Table 5). Of these, two isolates exhibited both wild-type (CAC) and mutant-type (TAC) alleles at codon 445 (Fig. 1E and F),four isolates showed double-point mutations, and two isolate had different base mutations at codon 445.

### Statistical analysis of detection performance

Based on the Kappa consistency test, the Kappa value between nucleotide MALDI-TOF MS and phenotypic DST was 0.91, indicating excellent agreement (Table 3). And the AUC for nucleotide MALDI-TOF MS detection was 0.96, further demonstrating its high diagnostic efficiency (Table 3).

## DISCUSSION

The rapid and accurate detection of drug resistance in M.tb is critical for effective TB control, especially in high-burden countries. Rif resistance, primarily caused by mutations in the *rpoB* gene, serves as a key marker for MDR-TB (3–5). In this study, we developed a novel nucleotide MALDI-TOF MS assay to detect RIF resistance. The optimized method used a single pair of PCR primers and 13 SBE primers, enabling the identification of 19 single-nucleotide mutations within six common RIF resistance-associated codons in the rpoB gene (Table 2) (3, 21). Compared to phenotypic DST and DNA sequencing, the nucleotide MALDI-TOF MS demonstrated high sensitivity (93.2%) and specificity (98.1%) for detecting rpoB mutations. Furthermore, this method showed excellent concordance with phenotypic DST (kappa value = 0.91) and DNA sequencing (93.4%), confirming its diagnostic accuracy and reliability. These findings are consistent with those of previous studies. For instance, Ou X et al. (17) reported sensitivity, specificity, and kappa values of 98.2%, 98.7%, and 0.97, respectively, for detecting RIF resistance using nucleotide MALDI-TOF MS. Similarly, Wu X et al. (19) observed sensitivity, specificity, and concordance rates of 92.2%, 100.0%, and 94.6%, respectively. These results collectively underscore the ability of nucleotide MALDI-TOF MS to rapidly, efficiently, and accurately detect rpoB mutations associated with RIF resistance, providing a valuable tool for improving the diagnosis of drug-resistant TB.

RIF, a critical first-line anti-TB drug, targets the RNA polymerase β subunit encoded by the *rpoB* gene. The detection of *rpoB* mutations is essential for identifying RIF resistance. Although the specific mutation sites may vary by geographic regions, studies have confirmed that 90%–98% of RIF resistance is caused by mutations within the 426 to 452 amino acid codons of *rpoB* (3, 24, 25). The most common mutations occur at codons 450 and 445, which are frequently associated with high-level resistance (e.g., Ser450Leu, His445Tyr, His445Asp, and Ser450Trp). In contrast, mutations at codons 430, 432–435, 440, 441, 448, and 452 typically confer intermediate or low-level resistance, while certain amino acid substitutions at codons 426–428, 436, 442, and 451 may not be associated with RIF resistance (26–28). By detecting mutation sites and the nature of mutations in drug resistance-related genes, clinicians can gain a better understanding of the drug resistance profiles in TB patients. In this study, we designed a single pair of PCR primers to amplify the *rpoB* gene, specifically targeting the 81 base-pair (bp) RIF resistance-determining region (RRDR), which spans codons 426 to 452. The nucleotide MALDI-TOF MS assay facilitated high-throughput analysis, allowing for the examination of 1 to 384 samples per run, and successfully identified 14 *rpoB* mutation genotypes associated with RIF resistance. Notably, mutations at codons 450, 445, and 435 accounted for 84.5% of all detected mutations. The most frequent mutation site was found at codon 450 (54.4%), with the Ser450Leu (TCG→TTG) mutation being the predominant type (52.4%). These findings are consistent with those of global studies, where mutations at codons 445 (e.g., His445Tyr, CAC→TAC) and 435 (e.g., Asp435Val, GAC→GTC) have also been frequently reported (3, 17, 19, 21, 25). These results highlight the clinical significance of targeting codons 450, 445, and 435 in molecular diagnostics for RIF resistance.

In this study, nine isolates showed discordant results between nucleotide MALDI-TOF MS and phenotypic DST. Several factors may account for these discrepancies. First, the nucleotide MALDI-TOF MS assay detects only known RIF resistance-associated mutations and may fail to identify rare mutations, novel variants, or mutations outside the 81 bp RRDR. Additionally, other mechanisms of RIF resistance, such as alterations in drug efflux pumps, membrane permeability, or enzymatic drug inactivation, could contribute to phenotypic resistance in the absence of detectable *rpoB* mutations (28, 29). Second, discrepancies may arise from limitations of the absolute concentration method using the L-J medium in phenotypic DST, which can produce false-positive or false-negative results. For example, phenotypic DST may not detect low-level resistance or heteroresistant populations (9, 19). Furthermore, three isolates with double *rpoB* mutations identified by DNA sequencing were reported as single mutations by nucleotide MALDI-TOF MS. This limitation occurs because the assay does not include all known RIF resistance-associated mutations, such as Gln429His (CAG→CAC), Leu430Arg (CTG→CGG), and Asn437His (AAC→CAC). Although uncommon, these mutations illustrate the genetic diversity underlying RIF resistance (30). Additionally, one isolate with a missense mutation(TAC) at codon 450 (TCG) showed discordant results between nucleotide MALDI-TOF MS and DNA sequencing. Synonymous mutations may lead to false-positive results. However, as a microsequencing technique, the nucleotide MALDI-TOF MS assay offers high accuracy and specificity by extending a single base, which reduces the likelihood of errors compared to traditional sequencing methods.

TB patients may exhibit heteroresistance due to factors such as infection from multiple sources, variations in treatment efficacy, or microevolution of bacterial subpopulations in different infection sites (10, 31–33). In this study, DNA sequencing confirmed heteroresistance in 14.3% (8/56) of RIF-resistant MTB isolates, a rate higher than that previously reported (4.8%, 8/168)(19). Notably, two isolates with coexisting wild-type and mutant alleles were particularly challenging for phenotypic DST (10). The ability of nucleotide MALDI-TOF MS to detect heteroresistance is a significant advantage, enabling clinicians to design better treatment regimens early and prevent delays in diagnosis that could exacerbate the spread of drug-resistant TB (19).

Despite its diagnostic potential, this study has several limitations. First, it focused exclusively on RIF resistance, and the applicability of nucleotide MALDI-TOF MS for detecting resistance to other anti-TB drugs requires further investigation. Second, although the sample size was adequate, it may not fully represent the spectrum of *rpoB* mutations across different geographic regions. Future studies should include larger and more diverse populations and evaluate the performance of nucleotide MALDI-TOF MS for detecting resistance to other first- and second-line anti-TB drugs.

In conclusion, nucleotide MALDI-TOF MS is a reliable, rapid, and accurate method for diagnosing RIF resistance-associated mutations in *M.tb*. Its high concordance with DNA sequencing, ability to detect heteroresistance, and capacity to provide rapid results make it a valuable tool for TB diagnostics. Future research should explore its application for detecting resistance to other anti-TB drugs and validate its use in diverse clinical settings.

## Data Availability

All data generated or analyzed during this study were included in this published article.
